# Deep learning enabled pseudonymization for preserving data privacy of financial identifiers in public documents in India

**DOI:** 10.1038/s41598-026-39309-6

**Published:** 2026-02-10

**Authors:** R. Roopalakshmi, Saurabh Kailas, R. Sreelatha

**Affiliations:** 1 School of Computer Engineering, Manipal Institute of Technology, Manipal Academy of Higher Education (MAHE), 576104 Manipal, Karnataka India; 2 Department of ISE, B.M.S. College of Engineering, Bangalore, Karnataka India

**Keywords:** Pseudonymization, CNN, Handwritten Signatures, Public-domain Documents, SuperPoint Architecure, ORB, FAST, SIFT, ResNet., Image processing, Machine learning, Data publication and archiving, Communication and replication, Data integration

## Abstract

The increasing digitization and transmission of government-issued electronic documents have intensified the need to protect the ’Handwritten signatures’-recognized as ’critical biometric identifiers’ from identity-related data breaches. For instance, as per 2025-RSA ID IQ Report, 40% of respondents reported Identity-related data breaches and 66% emphasized the significant damages caused by these breaches to their organizations. The existing privacy-preserving anonymization research is primarily focusing on facial features and fingerprints, whereas the Pseudonymization of handwritten signatures in publicly accessible documents remains largely underexplored in the literature. This research study proposes a new Fully Convolutional Neural Network (CNN)-based Pseudonymization framework using SuperPoint architecture integrated with Differentiable output decoding, which aims to identify and pseudonymize the handwritten signatures in public-domain documents, specifically in Indian Government issued Permanent Account Number (PAN) cards. In contrast to traditional anonymization approaches, this pseudonymization technique preserves document utility by securing sensitive data and thereby enables traceable identity protection without compromising the structural integrity of input documents. Extensive evaluations are carried out on a curated dataset of 500+ real-world PAN cards, which establishes the model’s robustness and applicability in large-scale deployments. The results of comparative analysis with baseline techniques including ORB, FAST, SIFT and deep CNN, clearly demonstrate the superior performance of the proposed method in terms of various metrics such as Precision, Recall, SSIM, runtime efficiency, and spatial overhead. In addition, the research findings suggest practical implications for embedding CNN-based pseudonymization into Public-sector Document processing pipelines, which supports secure and utility-preserved digital archiving in compliance with modern privacy GDPR standards.

## Introduction

In this digital world, the Identity-related data breaches are more severe and costing more for organizations than any other kind of attacks. For instance, in the latest 2025-RSA ID IQ Report^1^ comprising the responses collected from 2000 tech professionals from 62 countries, highlights these key insights: 40% of respondents reported Identity-related security breaches and 66% reported that their organization is severely damaged because of these identity-related data breaches. Generally, three types of user information/identifiers are utilized in public domain documents, as given by: a) Biometric identifiers - include physiological features (e.g., face, iris, fingerprint) and behavioural traits (e.g., voice, gait, gesture); b) Non-biometric identifiers - include identifiers such as text, speech, dressing style and hairstyle; c) Soft biometric identifiers- include non-distinctive human features such as age, gender, tattoos, and eye color^2^-^3^. In general, Biometric identification systems employ individual’s unique physical features such as fingerprints, iris scan, and facial appearance for the verification process. In traditional methods user’s credentials such as password, PIN or smart card are utilized for authentication purposes, whereas in biometric recognition systems distinctive features of individuals are employed^4,5^. The biometric recognition systems are quite efficient in terms of avoiding unnecessary complex authentication processes, since they are rarely susceptible to human mistakes or theft. In addition, Biometric identifiers offer multiple benefits, when compared to conventional authentication methods as given by: a) They deliver an optimal user experience by naturally binding non-transferable and highly distinctive biometric features; b) They are less vulnerable to typical hacking kind of security attacks; c) They are resistant to theft, since it is extremely hard to duplicate or counterfeit it. Due to these core benefits, biometric-based authentication systems have gained huge attention in research domains such as security services and access control of both the private entities and government institutions in the past few decades^6^.

In general, Anonymization is defined as, the process of removing or modifying the personal identifiers so that the critical data is encrypted and restricted from exposure to unauthorized personnel or public access^7^. Specifically, the primary objective of anonymization is to protect the utility of the data as well as to protect the privacy of end users^8,9^. In data security and privacy context, anonymization is generally used to meet the privacy regulations and laws, which protect personal data against unauthorized usage or access. Despite of its widespread usage, the literature lacks a standardized definition of anonymization, which leads to certain ambiguities regarding its implementation and scope. Different sources offer varying definitions of anonymization and present multiple approaches for its practical implementation. For instance, Meden et al.^10^ explained anonymization as a method of hiding personal identifiers with suitable substitutions, thus ensuring that sensitive data is not revealed and not used for unrelated purposes for which it was collected. This definition focuses on ensuring that privacy of individuals is not compromised by preventing data from being traced back to any particular person. In contrast, Nelson et al.^11^ proposed the use of de-identification as a reversible process of concealing Personally Identifiable Information (PII) in personal records. The aim of de-identification, as defined by the authors is to minimize the risk of unintended identity data disclosures, while permitting the data to be utilized for some secondary purposes, including research or analysis.

The difference between anonymization and de-identification is considered as critical, depending upon the the security level needed in different contexts. Specifically, anonymization is generally referred as an irreversible process, which is designed to provide maximum privacy protection, whereas de-identification is implemented with re-identification possibilities and hence may become problematic if the data is leaked or accessed inappropriately^11^. These controversies reflect the challenges of developing clear standards and guidelines for data privacy domain, in which huge volumes of personal data are gathered and analysed. As data privacy issues are exponentially increasing, it is very much essential to establish a standardized process for anonymization and de-identification, which maintains a clear balance between privacy protection and data usage demands^11^. On the other hand, a more precise definition of these technologies are essential, specifically in Artificial Intelligence (AI) and Machine Learning (ML) domains, which pose additional complexities to data protection. AI and ML technologies are based on large datasets with sensitive data, and therefore, proper anonymization or de-identification of such data becomes even more important to avoid accidental privacy infringement or data breaches.

In this internet era, generally, the public domain documents of a user, such as a Facebook page, may include different types of biometric identifiers, which need to be de-identified to protect user’s privacy, popularly known as Multimodal De-identification process^12^. Research in the field of de-identification of non-biometric identifiers initially emerged in the context of medical applications, particularly in terms of text-based de-identification of patient information in Personal Health Records (PHR)^13^. In healthcare, it is crucial to maintain the confidentiality of patient’s personal details. One of the most popular method for achieving this confidentiality is, by replacing identifiable terms in clinical records with non-identifiable terms. This kind of replacement strategies decrease the risk of misuse and thereby facilitates the usage of protected data for research and scientific purposes^12^. Although extensive research is conducted on biometric de-identification methods, including face detection^10^ and gait recognition^11^, very few attempts are made towards the anonymization text-based sensitive identifiers such as handwritten signatures.

### Motivation and contributions

The growing emphasis on user privacy in the digital era, specifically concerning sensitive personal identifiers such as handwritten signatures, highlights the importance of developing effective data protection solutions^12,13^. Though state-of-the-art privacy-preserving anonymization research is mainly focusing on biometric identifiers such as facial features and fingerprints, the anonymization of direct identifiers such as handwritten signatures in publicly accessible documents are largely underexplored in the literature. On the other hand, in this digital era, government-issued documents containing handwritten signatures are digitized, stored, and hugely transmitted across platforms, which raises serious concerns about data privacy and identity theft. Specifically, the Handwritten signatures, as one of the primary sensitive biometric identifiers, are largely vulnerable to misuse if they become exposed. Although anonymization could decrease this risk, still it fails to support valuable document utilization. However, Pseudonymization, allows these sensitive identifiers to be replaced with synthetic or masked representations while preserving the traceability under controlled conditions and thereby facilitates both the privacy and usability benefits. The motivation behind this research study is to address these challenges, by developing Convolutional Neural Network(CNN)-based framework for identifying and pseudonymizing handwritten signatures in public domain documents. Specifically, the key contributions of this research article are listed as follows:

1. **A novel SuperPoint CNN-based framework combined with Differentiable Output Decoding**: This study proposes a new Pseudonymization algorithm using Fully CNN-based architecture-known as ’*SuperPoint*’^14^ integrated with differentiable output decoding for pseudonymizing the handwritten signatures present in the government-issued documents, which efficiently masks the identity and preserves the structural utility of these documents.
2. **First-of-its-Kind Pseudonymization for Indian Government issued ID cards**: To the best of our knowledge, this is *first initiative of its kind*, which focuses on pseudonymization of sensitive signatures present in Indian Government issued Permanent Account Number(PAN)^15^ card documents using SuperPoint CNN-based model, setting it apart from existing literature.
3. **Comparative Evaluation of CNN-based Pseudonymization against Baseline methods**: In this research study, comparative analysis of proposed CNN-based model Vs state-of-the-art techniques including ORB^16^, FAST^17^ and SIFT^18^ and Deep CNN models are carried out to highlight the effectiveness of the proposed pseudonymization task.
4. **Real-World Testing of Framework using a Curated Dataset**: This research study is evaluated on the extensive curated dataset collection of 500+ government-issued PAN Card documents with extricated handwritten signatures for experimental benchmarking and reproducibility, which makes it viable for large-scale use.
5. **Comprehensive Evaluation using several Key Performance Metrics**: The proposed pseudonymization technique is comprehensively evaluated using several key performance metrics including Precision, Recall, Localization Error, Structural Similarity Index Measure (SSIM) Score^19^, feature maps, space efficiency and runtime complexity by means of highlighting the complete pseudonymization pipeline of handwritten signatures.
6. **Recommendations for integrating CNN-based pseudonymization into Public-sector document processing pipelines**: The current study provides valuable insights into the role of CNNs in document privacy and thereby establishes a foundation for integrating biometric pseudonymization into secure data processing pipelines, which aligns with modern privacy regulations such as GDPR^9^. The research findings carry practical implications for digital archiving in the public sector, safeguarding identities and thereby ensuring secure document exchanges.

## Literature survey

The current literature on anonymization covers a broad range of problem domains including emphasis on data privacy in the medical field, particularly in terms of Personal Health Records (PHR)^20^. The primary objective of these studies is to ensure that sensitive information of patients are properly anonymized, so that unauthorized access or misuse can be prevented, while maintaining data utility for research and healthcare purposes. Another popular research problem in the existing anonymization literature is Gait Anonymization^21^, in which individual privacy is protected and also sufficient information for recognition is maintained. For example, Tieu et al.^21^ presented a Gait Anonymization technique using spatio-temporal generative adversarial networks (GANs) to anonymize gait data as well as to maintain important features for recognition. Further, anonymizing faces is also popular in the literature, which focuses on modifying facial features except facial expressions to ensure that the recognition of individuals is avoided while retaining the information required for targeted applications^22,23^.

In the field of biometric de-identification, Iris-based identification has gained more attention in the past decades^24^. For instance, Zhang et al.^24^ proposed a de-identification framework using Hough transformations, which are useful for maintaining privacy in scenarios where iris recognition is used. Research studies are carried out to focus on blurring or occluding tattoos in images while preserving the general content for privacy protection^25^. Recently, Pena et al.^26^ introduced a de-identification technique for facial expressions and the corresponding emotions so that facial recognition without violating user’s privacy can be achieved. Further, personality traits-based de-identification research^27^ is also introduced in the literature, which investigates the anonymization of personal traits, while still enabling meaningful analysis in social media and psychological studies. In^6^, the authors introduced a general model for constructing anonymous signatures in terms of combining benefits of anonymous credentials (AC) and group signatures(GS), which are applicable in the financial cryptographic domain.

Wu et al.^28^ introduced an object detection based technique, which can used to localize the handwritten regions from documents of Chinese and German languages. Sivaprasad et al.^29^ recently, proposed a deep-learning-based technique for classifying signatures on educational certificates, which can be used to improve the verification processes and also to maintain the integrity of academic credentials. Although, this technique achieved superior performance in distinguishing between genuine and forged signatures in educational certificates, yet it is less focused towards public-domain govt-authorized documents.

Latest research studies on data protection and anonymization mainly concentrates on text-based document data. For instance, Korytkowski et al.^30^ utilized fully CNNs-based method for removing sensitive data and preserving privacy in German language official documents . In^31^, the authors achieved anonymization of German financial documents by employing neural networks based models combined with contextual word representations. Recently, Hernandez et al.^32^ introduced AGORA system in a police context in Spain, which is used to recognize named entities and anonymize Spanish text documents. Van Rooji et al.^33^ proposed a Federated tool for anonymizing and annotating the image data related to Flicker logo dataset^34^, which used YOLOv5^35^ for model development. S’anchez et al.^36^ presented an automatic anonymization system for printed text documents of Spanish Language invoices.

Very Recently, in^37^, the authors presented a new biometric verification scheme, which utilizes online handwritten Random Digit String (RDS) for preserving user’s privacy during the process of online handwriting verification. Eberhardinger et al.^38^ presented a technique for automatically anonymizing images present in scanned documents including face, barcodes which reduces manual efforts and also ensures data protection compliance. Though this method outperforms copy reference and automatic detection approaches, yet it fails to focus on anonymization of handwritten signatures in official documents.

### Research gaps identified in the existing literature

The existing privacy-preserving anonymization studies are primarily focusing on biometric identifiers such as facial features, Iris and fingerprints, whereas the pseudonymization of sensitive identifiers such as handwritten signatures in public-domain documents are scarcely investigated in the literature. From another perspective, government-issued ID cards containing handwritten signatures are hugely transmitted across platforms, which raises serious concerns about data privacy and identity theft. Although anonymization could partially solve the vulnerability issues of these identity documents, yet it provides less support towards valuable document utilization. To tackle these issues, promising Pseudonymization framework, which replaces sensitive identifiers with masked representations, while preserving the traceability, privacy and ensuring usability benefits are very much essential in this digital era.

## Methods and materials

### Proposed methodology

**Fig. 1:**
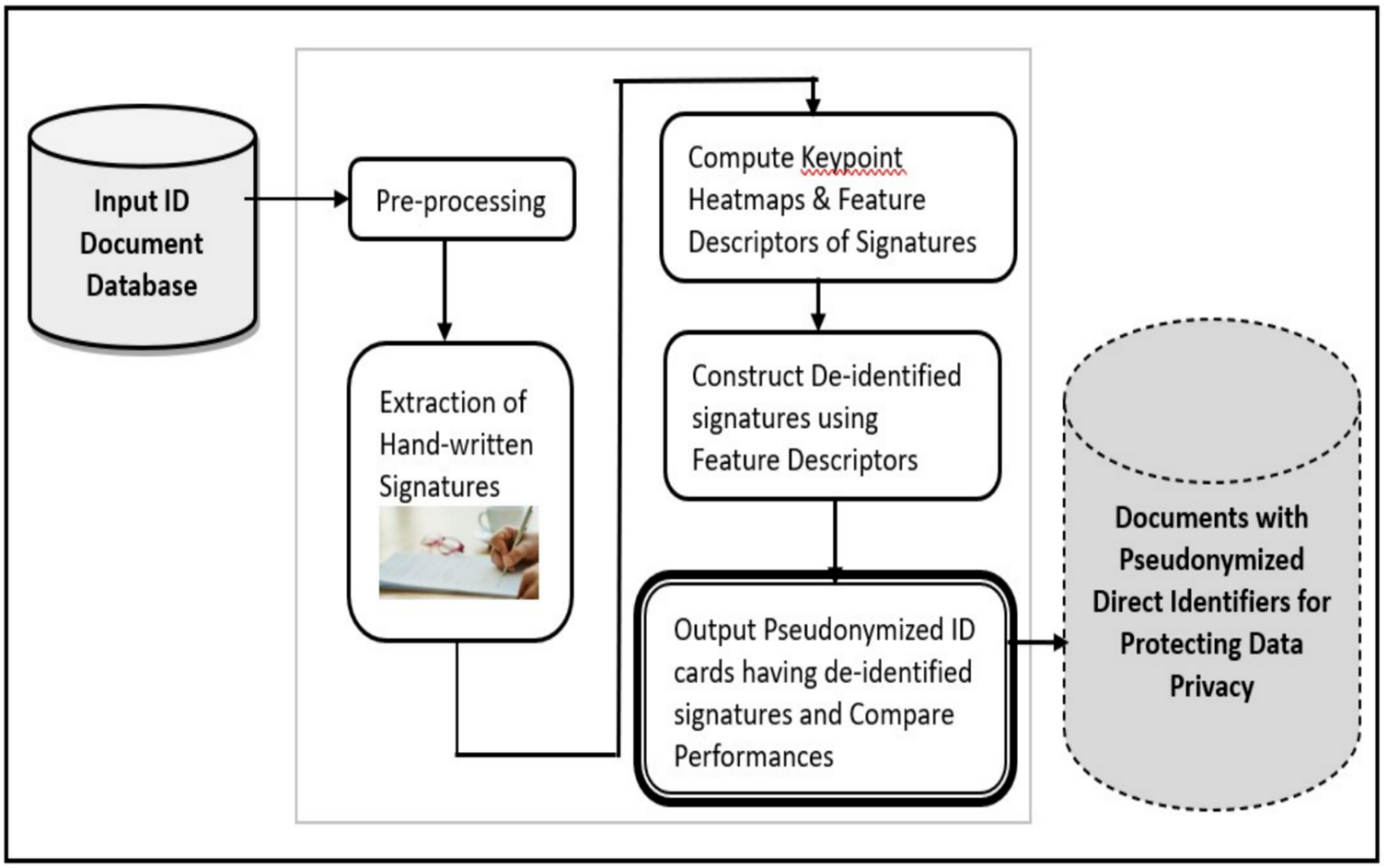
Blockdiagram showing Overview of the Proposed Pseudonymization Framework.

Figure 1 shows the blockdiagram of the proposed pseudonymization framework along with its submodules. In the proposed framework, initially the input PAN Card images are subjected to preprocessing, in order to enhance the quality of the pseudonymization task. Specifically, all the input images are resized to uniform scale across the dataset, which ensures consistency in image dimensions and also facilitates the subsequent analysis. Then, the coloured images are converted to gray-scale to reduce the computational complexity, since the pseudonymization seldom uses colour information. The resultant images are treated with Gaussian filters in the proposed framework, which smoothens the images and helps to remove unwanted irregularities in images. Followed by preprocessing, handwritten signatures are extracted from the input PAN Card images for achieving pseudonymization of these sensitive identifiers. Then, each signature image is processed using several feature descriptor techniques including ORB^16^, FAST^17^, SIFT^18^ and CNN-based methods to obtain the important features of signatures. Specifically, in each algorithm key-points are detected and descriptors are computed, which are indicated as colored circles over the signatures. Subsequently, pseudonymization is carried out to construct de-identified signatures, which masks the sensitive signature content while preserving the overall structure of the images. As a result, the output visualizations are generated by means of original signature region, the region with key-points marked, and the de-identified signature region, which allows for iteration-wise comparison and analysis across multiple images. In this way, the proposed pseudonymization methodology presents a systematic approach for protecting privacy in PAN card images while maintaining its usability for research and analysis purposes.

### Proposed methodology

In this work, we propose a fully convolutional pseudonymization framework, known as ’SuperPoint Architecture^14^’, for anonymizing the handwritten signatures. In general, SuperPoint architecture is a self-supervised deep learning model, which is designed to effectively detect keypoints and compute descriptors in general image processing applications^14^. Conventional CNN-based approaches primarily focus on feature extraction without explicit keypoint guidance, whereas our pseudonymization framework utilizes the SuperPoint backbone to achieve more discriminative and task-relevant feature representations, which is targeted for handwritten signature anonymization. Our framework adapts and extends the SuperPoint pipeline by integrating customized keypoint-descriptor modules and domain-specific training strategies and thereby allows the model to anonymize signature data while preserving essential structural information, which is essential for downstream verification. This methodological choice not only enhances the robustness of pseudonymization against re-identification attacks but also improves the utility of the transformed data for subsequent authentication tasks. In contrast to conventional CNN architectures, the proposed SuperPoint CNN-based pseudonymization framework integrates several core design components for enhancing anonymization performance and generating more discriminative feature representations, as detailed below:

- $$\star$$
  **Utilization of Fully Convolutional SuperPoint Architecture for Pseudonymization:** Unlike conventional CNNs, our framework utilizes fully convolutional architecture and thereby preserves spatial structure throughout the network, which is essential for achieving accurate keypoint-based anonymization of signatures. Further, our framework uses only convolutional layers and avoids the dimensionality collapse caused by dense layers in standard CNNs.
- $$\star$$
  **Joint Learning of Keypoints and Descriptors:** Traditional feature descriptors (e.g., SIFT, SURF) treat keypoint detection and descriptor computation separately, whereas proposed framework integrates these tasks in a single forward pass through a shared encoder with dual decoder heads (Detector and Descriptor heads respectively) and thereby scores better efficiency and robustness even under blur, occlusion, and viewpoint change conditions.
- $$\star$$
  **Data-Driven and Robust Against Input Variability:** In contrast to traditional gradient-based descriptors that fail in challenging conditions, our model employs deep learning architecture to learn discriminative and noise-resilient representations, which makes it suitable for real-world pseudonymization problem where handwritten signatures significantly vary in scale, rotation, and quality.
- $$\star$$
  **Kornia-Based Differentiable Decoding for Precision:** We incorporated Kornia^39^ for decoding network outputs into precise keypoints, descriptors, and affine frames, which enhances adaptability for variable input sizes and thereby ensures accurate feature mapping for this pseudonymization task.
- $$\star$$
  **Addressing the Real Problem of Pseudonymization:** The core problem in practical pseudonymization scenarios is ensuring privacy without losing structural integrity for downstream verification or analytics, especially under challenging conditions such as low-quality scans, distortions, or partial signatures. Our framework solves this by producing robust keypoint-based feature maps instead of storing identifiable pixel-level information. This ensures privacy, efficiency, scalability, and makes the solution deployable in real-world financial and legal domains where signature-based authentication is widely used.

These design elements establish the proposed framework as a specialized solution for privacy-preserving pseudonymization of handwritten signatures, which differs from the conventional hierarchical cascade of CNN layers. To rigorously evaluate its effectiveness, we performed a comprehensive comparative analysis of the proposed model and widely used traditional feature descriptors (ORB, FAST, and SIFT) and deep CNN models. The evaluation are carried out across multiple performance dimensions, including precision, recall, localization error, SSIM score, feature maps, space efficiency, and runtime complexity. These experiments were designed to quantitatively assess the framework’s anonymization capability and computational efficiency, which are illustrated in the Results and Discussion section.

#### SuperPoint network architecture used in our model

The proposed SuperPoint architecture outputs a heatmap of keypoints and dense descriptors. Specifically, our model employs a single Shared Encoder, which is the Convolutional backbone for achieving dimensionality reduction and feature extraction from the input image. The resultant features from the encoder are inputted into two decoder heads: Interest point Detector and Descriptor heads. The Detector head processes the features and displays keypoint heatmaps whereas the Descriptor head computes dense interest points and displays descriptor maps respectively. Precisely, the Layer-wise specifications of SuperPoint-based model architecture used in this study is illustrated in Table 1.

**Table 1:** SuperPoint Network Architecture Used in Our Model.

Stage	Layer	Output	Kernel	Stride	Padding	Activation
		Channels	Size			
Input	Grayscale	1	-	-	-	-
Block 1	Conv1a	64	3$\times$3	1	1	ReLU
	Conv1b	64	3$\times$3	1	1	ReLU
	MaxPool	-	2$\times$2	2	-	-
Block 2	Conv2a	64	3$\times$3	1	1	ReLU
	Conv2b	64	3$\times$3	1	1	ReLU
	MaxPool	-	2$\times$2	2	-	-
Block 3	Conv3a	128	3$\times$3	1	1	ReLU
	Conv3b	128	3$\times$3	1	1	ReLU
	MaxPool	-	2$\times$2	2	-	-
Block 4	Conv4a	128	3$\times$3	1	1	ReLU
	Conv4b	128	3$\times$3	1	1	ReLU
Heads	Detector	65	1$\times$1	1	0	-
	Head					
	Descriptor	256	1$\times$1	1	0	-
	Head					

**Fig. 2:**
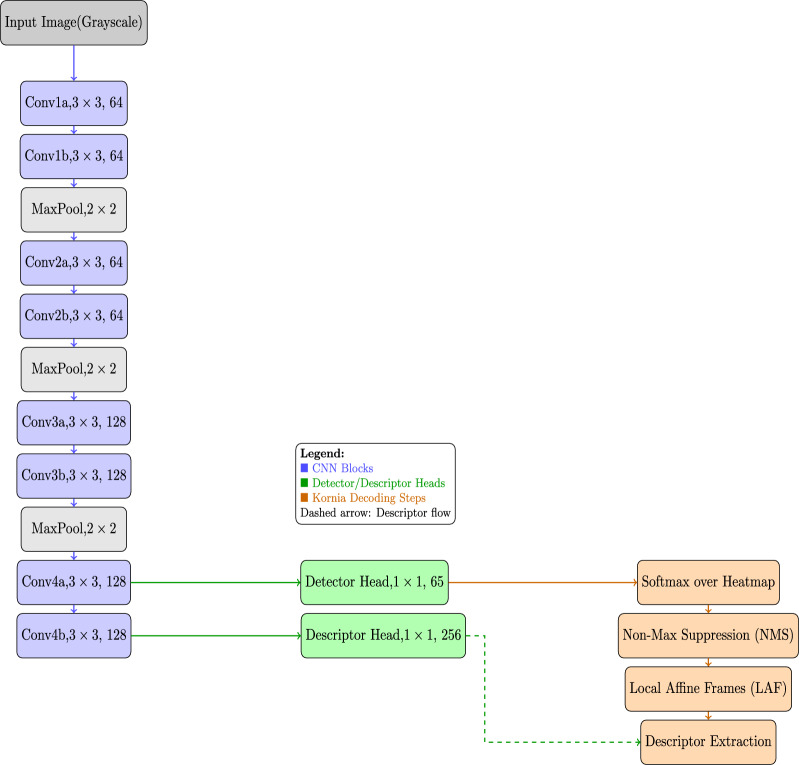
Detailed Architecture of Proposed Pseudonymization Framework.

The detailed architecture of our model using SuperPoint CNN integrated with Kornia-based decoding is illustrated in detail in Fig. 2.

#### Kornia based decoding of outputs

In this model, we employed *Kornia*^39^, a differentiable computer vision library available in PyTorch, which decodes the network’s raw outputs into actual keypoints, descriptors and local affine frames. Specifically, the step-by-step Kornia decoding employed in our model is detailed in Table 2.

**Table 2:** Kornia Based Decoding of Outputs.

Step	Function/Operation	Description
1	Softmax Function	Converts Detector head outputs
		into Keypoint Heatmaps.
2	Heatmap-to-Keypoints	Applies Non-Maximum Suppression, performs thresholding
	and Keypoint Thresholding	and selects top keypoints.
3	Compute LAF	Computes Local Affine Frames (LAF), which
		represents keypoint position, scale, and orientation.
4	Collect Descriptors	Extracts dense descriptors for detected keypoints
		from the Descriptor head output.
5	Returns Dictionary	Returns dictionary ({keypoints, descriptors,
		LAF, scores}) for using in the model.

Algorithm 1 illustrates the Pseudonymization algorithm utilized in the proposed system in the form of step-by-step procedure.

**Algorithm 1:**
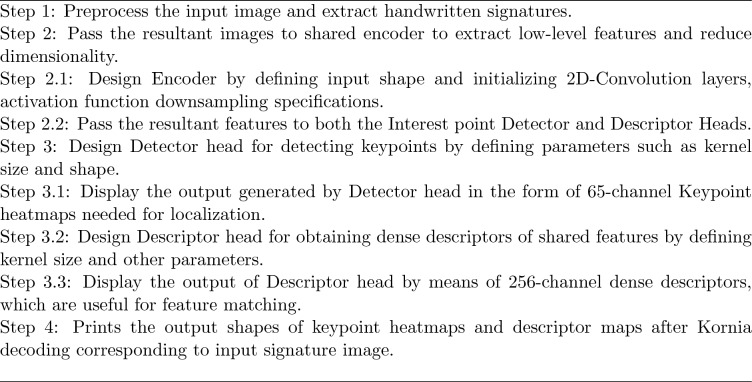
SuperPoint CNN-Based Pseudonymization Algorithm

### Materials/Experimental setup

#### Database description

**Fig. 3:**
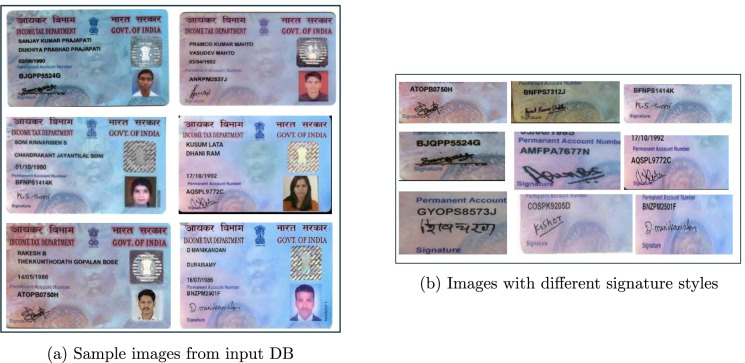
Sample snapshot showing PAN Card images and extracted signatures.

The experimental database of this research study consists of a collection of PAN Card images, which are Indian government-issued identification documents, primarily used for financial transactions and Income tax-related purposes^15^. Specifically, the PAN Card database of size 500+ images is constructed by collecting data from four popular open-access sources including kaggle datasets^40–43^. Specifically, the input database consists of wide range of PAN Card images, spanning from year 2008 to 2017 in different formats and backgrounds. Each PAN Card image includes personal details such as the individual’s name, photograph, PAN number, date of birth, signature, and a unique barcode. The average dimension of the images is 540$\times$405 pixels and the images showcase a wide range of human signature styles. Figure 3a presents the sample snapshot of PAN card images extracted from the input document database. Figure 3b highlights only the handwritten signatures extracted from the snapshot images of Fig. 3a.

It is evident from Fig. 3b, that there exist considerable amount of variations in handwritten signatures, in terms of stroke patterns, lengths, curves, and sizes, which pose significant challenges for the pseudonymization task.

The specifications of the system used for evaluating the pseudonymization accuracy of our model is illustrated in Table 3.

**Table 3:** Experimental Setup: Machine Specifications.

**Component**	**Specification**
CPU	APPLE M4 10-core
GPU	Integrated APPLE GPU 10-core (24 GB VRAM)
RAM	16 GB
Storage	512 GB
Operating System	Mac OS (64-bit)
Framework	PyTorch 2.0, TensorFlow and Keras
Programming Language	Python 2.7.16

#### Performance assessment metrics

To evaluate the accuracy of pseudonymization process carried out by the proposed framework, we employed several Performance Assessment metrics:

**Structural Similarity Index Measure (SSIM):** SSIM Score^19^ and its feature maps are employed in our model for evaluation purposes. Specifically, SSIM is a widely used perceptual metric to measure the similarity between two images. The conventional similarity metrics such as MSE (Mean Squared Error) considers difference in values, whereas SSIM utilizes luminance, contrast and structural changes in images, so that it is more aligned with the visual perception of humans. The Structural Similarity Index (SSIM) between two images $x$ and $y$ is calculated as follows:

1 $$\begin{aligned} \text {SSIM}(x, y) = \frac{(2\mu _x \mu _y + C_1)(2\sigma _{xy} + C_2)}{(\mu _x^2 + \mu _y^2 + C_1)(\sigma _x^2 + \sigma _y^2 + C_2)} \end{aligned}$$

where $\mu _x$, $\mu _y$ indicate the means of x and y, $\sigma _x^2$, $\sigma _y^2$ represent the variances of x and y, $\sigma _{xy}$ indicate the covariance of x and y and $C_1$ ,$C_2$ are constants.

**Precision:**Precision measures the proportion of correct positive predictions out of all predictions, which are classified as positive as given by,

2 $$\begin{aligned} \text {Precision} = \frac{\text {True Positives (TP)}}{\text {True Positives (TP)} + \text {False Positives (FP)}} \end{aligned}$$

**Recall:**Recall, also known as Sensitivity measures the proportion of actual positives, which are correctly predicted by the model as given by,

3 $$\begin{aligned} \text {Recall} = \frac{\text {True Positives (TP)}}{\text {True Positives (TP)} + \text {False Negatives (FN)}} \end{aligned}$$

**Localization Error:**Localization Error indicates the error in predicting the location/spatial position of an object in a keypoint-based task. It measures how far the predicted position/keypoint is from the ground truth position, as given by,

4 $$\begin{aligned} \text {Localization Error} = \sqrt{(x_p - x_g)^2 + (y_p - y_g)^2} \end{aligned}$$

where $(x_p,y_p)$ is the predicted point and $(x_g,y_g)$is the ground truth point respectively and Euclidean distance metric is employed.

## Results and discussion

This section demonstrates the evaluation results of the proposed framework in achieving pseudonymization of handwritten signatures present in sensitive PAN Card images. Specifically, CNN-based SuperPoint architecture is employed in this study for anonymizing the sensitive identifiers in the publicly available documents, as described in Section 3. Further, the performance comparison of proposed framework with the state-of-the-art feature descriptors including SIFT, FAST, ORB are elaborated in the upcoming subsections.

### Pseudonymization pipeline of proposed model

**Fig. 4:**
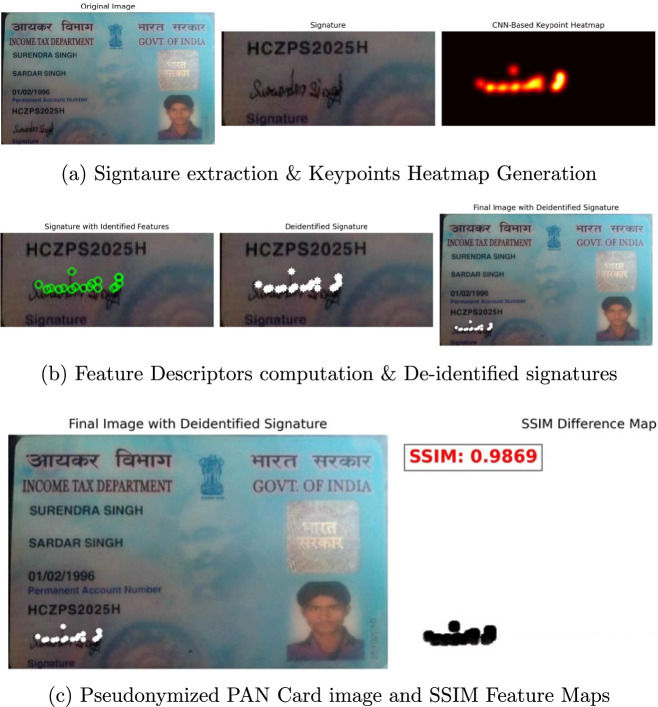
Pseudonymization Pipeline of Signatures using SuperPoint Architecture.

Figure 4 illustrates the implementation of Proposed Pseudonymization Pipeline on PAN Card signatures using SuperPoint CNN-based architecture, which specifies stage-by-stage process in left to right manner. Specifically, Fig. 4a describes the output of Detector head module of SuperPoint architecture in the form of keypoints heatmap as mentioned in Section 3. More specifically, the leftmost image in Fig. 4a indicates the ’*Original Input image*’ - which represents the unprocessed PAN card image comprising the original handwritten signature. The middle ones in Fig. 4a represents the handwritten signatures extracted by the proposed framework for proceeding with the pseudonymization process. The rightmost image in Fig. 4a clearly shows the CNN-based Keypoints Heatmap generated by the Detector head in the form of ’*yellowish orange*’ colored maps. Further, CNN-based heatmap shown in Fig. 4a clearly highlights almost all the significant keypoints within the signature, which are detected as salient handwriting features by the proposed framework.

Figure 4b shows the process of computation of feature descriptors followed by the pseudonymization of signatures in the form of 3 output images respectively. Specifically, the leftmost image in Fig. 4b indicates the dense descriptors computed by the Descriptor head of the CNN model in terms of ’*Green colored circles*’. It can be observed from Fig. 4b leftmost image that, the green circles marked on the extracted signature covers almost all the critical features, which are essential for carrying out the pseudonymization process. The middle image in Fig. 4b represents the output of pseudonymization in the form de-identified signatures, which are indicated in ’*White colored circles*’. It is evident from this figure that, the identified keypoints are sufficiently masked to achieve pseudonymization of handwritten signature, so that the overall appearance is also preserved. The rightmost image in Fig. 4b shows the Updated PAN Card Image with De-identified Signatures, in the form of masked identifiers to protect the integrity of the document.

Figure 4c demonstrates the efficiency of the proposed framework in terms of SSIM score and feature map respectively, which clearly presents the visual similarity between the original and pseudonymized signatures of input images. Figure 4b and c represent the outputs, which shown separately to explicitly highlight the SSIM feature map and the corresponding SSIM score for the given input image. Precisely, the left image in Fig. 4c shows the output of proposed CNN-based architecture by means of Final PAN Card image with de-identified signatures in *’white colored’* circles. The right image in Fig. 4c shows the SSIM score of 98.69% in *’Red colored boxed text’* at the top portion and SSIM feature maps in ’*Black colored circles*’ at the bottom portion. The SSIM score of 98.69% indicates near-perfect similarity, which emphasizes that the sensitive signature is efficiently anonymized with minimal visual distortions to the rest of the document. Further, the highest SSIM scores and feature maps demonstrate the efficiency of the proposed pseudonymization system, in terms of de-identifying the sensitive signatures, without distorting the overall visual fidelity of PAN Card images.

### Performance analysis of the proposed framework

**Fig. 5:**
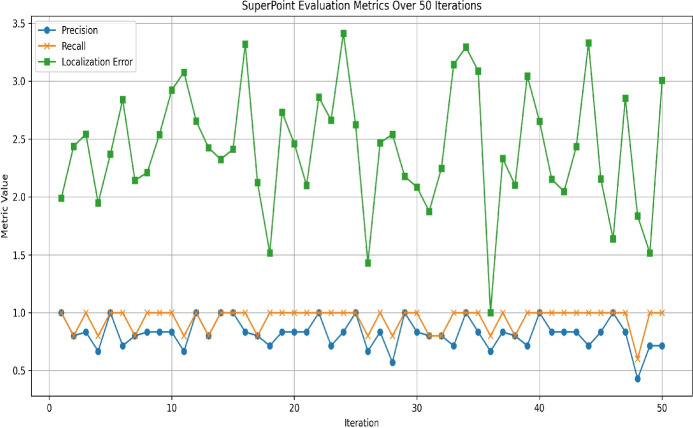
Accuracy analysis: Precision, recall, and Localization Error Metrics.

Figure 5 presents the performance analysis of the proposed Pseudonymization framework by means of Precision, Recall, and Localization Error metrics, which are calculated across 50 training iterations. Specifically, Precision results of the proposed framework is indicated in ’*Blue colored Line*’ with Circle Markers, which measures the proportion of correctly identified keypoints Vs all the detected keypoints. Although few dips can be observed in Fig. 5 plot, still the Precision stays between 0.6 to 0.9 across iterations, which proves the consistency of the proposed system. The Recall results of the proposed pseudonymization framework is shown in ’*Orange colored Line*’ with X Markers, which measures the proportion of correctly identified keypoints Vs all the true feature descriptors. Although minor dips are visible in Fig. 5 plot, yet the Recall values are consistently high-close to 1.0, across iterations, which demonstrates the notable performance of the proposed CNN-based system in the given pseudonymization task. Figure 5 plot indicates the Localization Error in *Green colored Line* with Square Markers, which reflects the average distance error between the detected as well as actual keypoints. In Fig. 5 plot, the localization error varies from 1.5 to 3.5 with frequent peaks and dips and suggests slightly lesser accuracy, which can be fine-tuned by employing suitable post-processing techniques.

### Performance comparison with baseline techniques

To validate the effectiveness of proposed SuperPoint CNN-based framework for pseudonymization, a comprehensive performance comparison against different conventional techniques are conducted. Specifically, the following three baseline techniques are selected for comparison, as given by:

1. Oriented FAST and Rotated Brief (ORB) Features^16^,
2. Features from Accelerated Segment Test (FAST) Features^17^ and
3. Scale-Invariant Feature Transform (SIFT) Features^18^.

#### Pseudonymization of signatures using ORB features

ORB combines the FAST keypoint detector and the BRIEF descriptor with orientation compensation and scale invariance for identifying significant feature descriptors in an image^16^. To achieve pseudonymization of handwritten signatures using ORB feature descriptors, OpenCV library is utilized in the proposed framework. Specifically, ORB key-points are extracted by utilizing different key parameters such as maximum number of features, the scale factor, and the number of pyramid levels. The maximum number of features- parameter specifies the max no. of key-points that needs be detected by the ORB detector. Although higher this value detects more no.of key-points, still results in longer computations and huge memory usage. Due to these reasons, this maximum number of features is set to 100, after multiple trials. The scale factor determines the pyramid scale factor, which is used to create the image pyramid for multi scale feature detection. In general, the image pyramid is a hierarchical representation of the image at various different levels, such that scale factor >1 indicates downscaled image, whereas scale factor <1 indicates upscaled image. Typically, a value between 1.2 and 2 is chosen in this research study, after testing various values. Further, the number of pyramid levels specifies the number of levels, which is set as 4, after trying multiple thresholds.

**Fig. 6:**
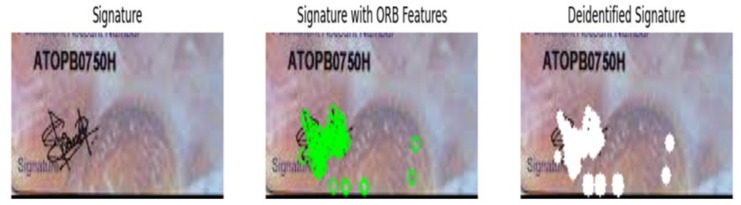
ORB features based pseudonymization.

After setting up the various parameters of ORB detector, it is applied to the image and the key points are identified, and their descriptors are computed. The algorithm first identifies all the potential key points in each signature image, followed by the computation of resultant feature descriptors. Figure 6 shows the output of pseudonymization process carried out using ORB features in three consecutive images respectively. Specifically, the leftmost image in Fig. 6 represents the signatures extracted from the input PAN Card images. The middle ones indicate the keypoints detected by ORB features in ’*Green colored circles*’, which represent the distinctive characteristics of signature images. After the detection of salient feature descriptors of signatures, pseudonymization process is carried out, which is indicated in ’*White colored circles*’ in Fig. 6. It can be observed from Fig. 6 that, the efficiency of ORB in this pseudonymization process is slightly lesser, due to the detection of more no. of outliers. The limited robustness of ORB detectors towards illumination changes might be the reason for this lower performance of ORB feature descriptors.

#### Pseudonymization of signatures using FAST features

**Fig. 7:**
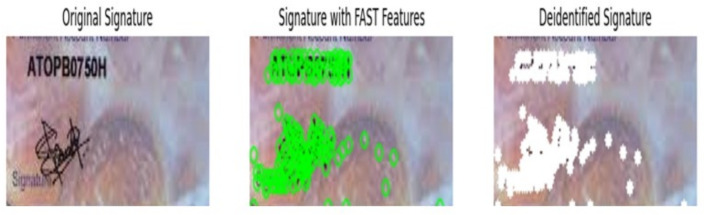
FAST features based pseudonymization.

Features from Accelerated Segment Test (FAST) is a corner detection algorithm, which is widely popular in the computer vision domain^17^. In the proposed framework, pseudonymization of signatures is carried out using FAST feature detectors by utilizing OpenCV library modules. In FAST algorithm, only keypoints are detected without the description of features, usage of a separate descriptor algorithm is essential for feature matching kind of applications. Due to these aspects, in the proposed framework, FAST is combined with BRIEF binary descriptor for implementing the feature description task. Figure 7 shows the output of pseudonymization process carried out in the proposed framework using FAST features in the form of three consecutive images. Specifically, the leftmost ones in Fig. 7 highlights the signatures extracted from the input PAN Card images. The middle ones in Fig. 7 clearly shows the significant keypoints detected by FAST technique in ’*Green colored circles*’, which consists of salient features of signature images. Once, the resultant salient feature descriptors of signatures are detected using FAST, the pseudonymization process is carried out and the results are indicated in ’*White colored circles*’ in Fig. 7. It is evident in Fig. 7 that, the accuracy of FAST technique in this pseudonymization process is slightly inferior, due to the detection of more insignificant interest points. Sensitive to noise,lighting conditions and Weaker in low-texture areas- might be the reasons for this lower performance of FAST feature descriptors in this pseudonymization task.

#### Pseudonymization of signatures using SIFT features

**Fig. 8:**
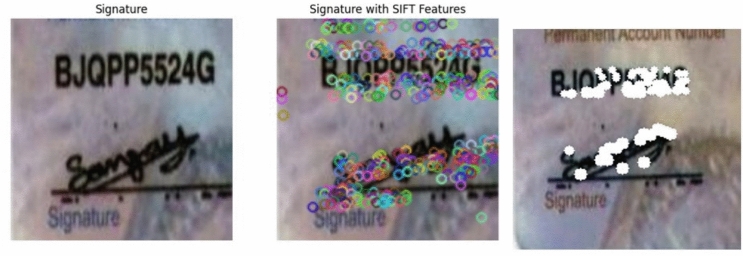
SIFT features based pseudonymization.

Scale-Invariant Feature Transform(SIFT) Feature detection and description algorithm^18^ is popularly used in computer vision domain from the past decades. SIFT detects keypoints using the Difference of Gaussians (DoG) across multiple scales followed by the assignment of orientations based on local gradient directions. SIFT describes each detected keypoint by means of a 128-dimensional vector with the help of gradient histograms. Figure 8 indicates shows the output of pseudonymization process conducted in the proposed framework using SIFT features in the form of three consecutive images. Specifically, the leftmost ones in Fig. 8 presents the signatures extracted from the input PAN Card images. The middle ones in Fig. 8 clearly indicates the important keypoints detected by SIFT approach in ’*Multi colored circles*’, which represent salient features of signature images. After the detection of salient feature descriptors of signatures using SIFT, the pseudonymization process is executed in the proposed framework and the corresponding results are indicated in ’*White colored*’ squares in Fig. 8. It is visible in Fig. 8 that, the accuracy of SIFT technique in this pseudonymization process is slightly lower in terms of detecting higher proportion of less discriminative keypoints and thereby overall performance is reduced. Limited robustness in moderately illuminated regions might be one of the reason for the lower scores of SIFT feature descriptors in this pseudonymization activity.

### Performance comparisons with baseline techniques

To demonstrate the performance of the proposed framework in this pseudonymization task, a comparative analysis is conducted against the state-of-the-art techniques including ORB^16^, FAST^17^ , SIFT^18^ and ResNet-18^44^ across multiple criteria, as presented in Table 4. Precisely, the performance results of proposed pseudonymization framework is compared in terms of different evaluation criteria including average no.of keypoints, average processing time, runtime efficiency, space efficiency and average SSIM score as indicated in Table 4.

**Table 4:** Performance of Proposed Vs Baseline Techniques.

Evaluation	ORB	FAST	SIFT	ResNet-18	Proposed
Criteria					
Average No. of	90.6	237.6	159.47	NA (Dense feature maps)	**126.81**
Keypoints / image					
Average processing	0.0033	0.0004	0.0121	0.0231	**0.0183**
time / image (s)					
Descriptor Size	256 bits	–	128 floats	512 floats	–
Runtime Efficiency Formula	$O(N \times D \times C)$	*O*(*N*)	$O(k \times N)$	$O(N \times F \times L)$	$O(k \times N)$
	*N*: No. of pixels	*N*: No. of pixels	*k*: No. of scales	*N*: No. of pixels	*k*: No. of scales
	*D*: Descriptor size		*k*: No. of scales	*F*: No. of filters	*N*: No. of pixels
	*C*: Comparisons			*L*: No. of layers	
Runtime Efficiency (Expanded)	$O(N \times 128 \times C)$	*O*(*N*)	$O(20 \times N)$	$O(N \times 512 \times 18)$	$O(127 \times N)$
Space Efficiency	$O(90.6 \times 128 \times N)$	$O(237.6 \times N)$	$O(159.47 \times 128 \times N)$	$O(\text {Feature maps} \times L)$	$O(126.81 \times 256 \times N)$
Average SSIM score (%)	81.92	79.37	89.56	95.15	**97.26**

As indicated in Table 4, FAST algorithm detects the highest average number of keypoints/image, which are redundant or less discriminative, due to its aggressive corner detection aspects. When compared to FAST technique, SIFT detects a moderately high number of keypoints, which are more distinctive due to its robustness capabilities. Although ORB detects the fewest keypoints and reduces computational burden, yet it might be missing the finer details. On the other hand, the SuperPoint CNN-based proposed system, detects fewer keypoints, when compared to others, and it manages retain higher-quality relevant features. Although the average processing time of proposed framework is slightly higher compared to baseline methods, due to model training aspects, but it results in extraction of more distinctive features of signatures. Table 4 evaluation results demonstrate that, the proposed method maintains a clear balance between keypoint quality and quantity by means of detecting a reasonable number of informative features, despite the modest increase in computational time.

It is evident from Table 4 results that, the average processing time of ORB is better than SIFT, while in terms of runtime efficiency, SIFT is better than ORB. This difference arises because average processing time and runtime efficiency capture different aspects of performance. Average processing time refers to the practical execution time for processing a single image on the given hardware, for which ORB scores lower than SIFT. The reason is, it uses binary descriptors, which reduces computational complexity per image. In contrast, runtime efficiency refers to the algorithmic complexity of descriptor matching process on large datasets. SIFT is slower per image, yet its asymptotic complexity of is O(k$\times$N), which results in higher efficiency in theoretical large-scale scenarios. Thus, ORB is faster for individual image processing, while SIFTs theoretical complexity scales better under descriptor matching conditions.

It can be observed from Table 4 that, FAST detector is the most computationally efficient with linear complexity, whereas ORB technique is slightly more complex due to the computation of feature descriptors for each keypoint. Although, SIFT and the proposed method involve multi-scale processing, still the proposed framework employs more scales for enhancing feature robustness and thereby results in higher computational cost. In case of space efficiency, FAST algorithm has the lowest value, due to the absence of descriptor computations, whereas SIFT has the highest value due to the generation of dense and high-dimensional descriptors. However, the proposed pseudonymization framework maintains a perfect balance between keypoint quantity and descriptor size and thereby ensures space efficiency and preserves overall visual appearance of signatures.

It is evident in Table 4 results that, although ResNet-based models demonstrate strong semantic representation capability, they typically yield a lower SSIM score when compared to the Proposed model. ResNet-18 Architecure is optimized to learn high-level semantic features rather than preserving fine-grained pixel structures, due to which, it resulted in slightly lower SSIM scores. The proposed SuperPoint CNN-based pseudonymization technique significantly outperforms the baseline methods by achieving 97.26% score and thereby demonstrates its better feature representation and structural preservation capabilities.

## Conclusion and future work

This research study presents a CNN-based Pseudonymization framework by employing superPoint architecture for pseudonymizing handwritten signatures in Indian Govt-issued Permanent Account Number(PAN) card documents. This study suggests practical guidelines for embedding CNN-based pseudonymization into public-sector DBMS pipelines, which supports secure and utility-preserved digital archiving. In future, the proposed framework can be extended to different languages and various govt-authorized identity documents by means employing complex deep learning models along with suitable optimization strategies.

Although the present study focuses on a fully CNN-based pseudonymization framework, in future this study can be advanced by integrating more contemporary deep learning paradigms. One promising direction is the incorporation of transformer-based architectures, such as Vision Transformers (ViT), Swin Transformers, or hybrid CNN–Transformer backbones, which proved their superior capability in modeling long-range spatial dependencies and global context when compared to purely convolutional designs.

Another promising direction is the development of lightweight and efficiency-oriented architectures which are tailored for real-world pseudonymization framework deployment. Specifically, techniques such as depth-wise separable convolutions, dynamic token pruning, and knowledge distillation can be explored, which may reduce computational complexity while preserving performance of the model. The integration of efficient transformer variants (such as MobileViT, DeiT, or TinyViT) and neural architecture search (NAS) based lightweight models could enable real-time pseudonymization on edge devices with limited resources. Further, combining these advancements with domain-specific optimization strategies such as attention-based interpretability modules, and differential privacy techniques on larger signature databases, might result in a next-generation pseudonymization framework, which is both highly secure and practically deployable in public-sector digital archiving pipelines.

## Data Availability

This study used open-access datasets, which can be found on, 1) https://universe.roboflow. com/jainam-chhadwa/pan-card-7nydd/dataset/1, 2) https://www.kaggle.com/datasets/ gunhcolab/pan-card, 3) https://www.kaggle.com/datasets/sparsh2002/pan-card-dataset, 4) https://www.kaggle.com/datasets/sparsh2002/govtiddataset. The curated dataset, additional materials or source code supporting the conclusions of this article will be made available by the authors on request.
